# COMMD1, from the Repair of DNA Double Strand Breaks, to a Novel Anti-Cancer Therapeutic Target

**DOI:** 10.3390/cancers13040830

**Published:** 2021-02-16

**Authors:** Amila Suraweera, Pascal H. G. Duijf, Christian Jekimovs, Karsten Schrobback, Cheng Liu, Mark N. Adams, Kenneth J. O’Byrne, Derek J. Richard

**Affiliations:** 1 School of Biomedical Sciences, Centre for Genomics and Personalised Health, Translational Research Institute, Queensland University of Technology (QUT), 37 Kent Street, Woolloongabba, QLD 4102, Australia; pascal.duijf@qut.edu.au (P.H.G.D.); c.jekimovs@hdr.qut.edu.au (C.J.); k.schrobback@qut.edu.au (K.S.); mn.adams@qut.edu.au (M.N.A.); k.obyrne@qut.edu.au (K.J.O.); 2 Princess Alexandra Hospital, 199 Ipswich Road, Woolloongabba, QLD 4102, Australia; 3 Centre for Data Science, Queensland University of Technology (QUT), Brisbane, QLD 4000, Australia; 4 University of Queensland Diamantina Institute, University of Queensland, Brisbane, QLD 4102, Australia; 5 QIMR Berghofer Medical Research Institute, 300 Herston Road, Herston, QLD 4006, Australia; John.Liu@qimrberghofer.edu.au; 6 Envoi Specialist Pathologists, 5/38 Bishop Street, Kelvin Grove, QLD 4059, Australia

**Keywords:** COMMD1, genomic stability, DNA double strand break repair, non-small cell lung cancer, novel therapeutic target

## Abstract

**Simple Summary:**

Lung cancer is the most commonly diagnosed cancer worldwide and additionally the most common cause of death from cancer, with non-small cell lung cancers (NSCLC) being the most commonly diagnosed form of the disease. As drug resistance is a key issue halting chemotherapy effectiveness, there is a great need to identify new therapeutic targets. The aims of this study were to investigate the function of the protein, COMMD1, in the repair of DNA double strand breaks and the therapeutic potential of COMMD1 in NSCLC. Here, we demonstrate for the first time how an additional COMMD family member, COMMD1, functions in the repair of DNA double strand breaks and may be relevant as a therapeutic target and prognostic factor in NSCLC. These novel findings highlight the potential of a novel approach to NSCLC therapy, by targeting an overexpressed protein.

**Abstract:**

Lung cancer has the highest incidence and mortality among all cancers, with non-small cell lung cancer (NSCLC) accounting for 85–90% of all lung cancers. Here we investigated the function of COMMD1 in the repair of DNA double strand breaks (DSBs) and as a prognostic and therapeutic target in NSCLC. COMMD1 function in DSB repair was investigated using reporter assays in COMMD1-siRNA-depleted cells. The role of COMMD1 in NSCLC was investigated using bioinformatic analysis, qRT-PCR and immunoblotting of control and NSCLC cells, tissue microarrays, cell viability and cell cycle experiments. DNA repair assays demonstrated that COMMD1 is required for the efficient repair of DSBs and reporter assays showed that COMMD1 functions in both non-homologous-end-joining and homologous recombination. Bioinformatic analysis showed that *COMMD1* is upregulated in NSCLC, with high levels of *COMMD1* associated with poor patient prognosis. *COMMD1* mRNA and protein were upregulated across a panel of NSCLC cell lines and siRNA-mediated depletion of COMMD1 decreased cell proliferation and reduced cell viability of NSCLC, with enhanced death after exposure to DNA damaging-agents. Bioinformatic analyses demonstrated that COMMD1 levels positively correlate with the gene ontology DNA repair gene set enrichment signature in NSCLC. Taken together, COMMD1 functions in DSB repair, is a prognostic maker in NSCLC and is potentially a novel anti-cancer therapeutic target for NSCLC.

## 1. Introduction

Lung cancer remains the leading cause of death from cancer as well as the most frequently diagnosed cancer globally [1,2,3]. In 2018, lung cancer accounted for 11.6% of the total cancer burden, with 2.1 million new diagnoses and 1.8 million deaths worldwide [4]. In the European Union, the lung cancer rate in 2020 is predicted to be 31.6 per 100,000 in men and 15.1 per 100,000 in women and estimated to account for approximately 20% of all cancer deaths [5]. There are two main forms of lung cancers, non-small cell lung cancers (NSCLC) which account for 85–90% of lung cancers and small cell lung cancers which account for 10–15% of lung cancers. There are three main types of NSCLC, adenocarcinoma (ADC), squamous cell carcinoma (SCC) and large cell carcinomas (LCC), which comprise roughly 40%, 30% and 10% of all lung cancers, respectively [6,7,8].Treatment options for NSCLC are stage specific, with complete surgical resection the best option for patients with stage I–II NSCLC, with a five-year survival rate of approximately 80–90% in stage IA and for stages IB, IIA and IIB consisting of approximately 73%, 65% and 56% five-year survival, respectively [9]. The majority of NSCLC patients present at advanced stage IIIB or IV, where the prognosis remains poor and relapse after surgery is observed. Indeed, the five-year survival of stage III patients is only 15% [10]. Radiotherapy remains a frontline treatment option for early-stage NSCLC sufferers not suitable for surgical resection [11].

Immunotherapy has revolutionized the treatment of NSCLC in recent years. To date, the FDA has approved five different immune checkpoint inhibitors; pembrolizumab (Keytruda), atezolizumab, durvalumab, ipilimumab and nivolumab for the treatment of metastatic NSCLC [12,13]. In addition to monotherapy with immune checkpoint inhibitors, at present there is an expansion of clinical trials combining immune checkpoint inhibitors with radiotherapy [14], platinum-based chemotherapy [15], different immunotherapies [16] and histone deacetylase inhibitors [17]. However, despite recent advances in immunotherapy and targeted therapies, the global five-year survival rate for NSCLC is still as low as 4–17% [3,9,18]. The identification of novel NSCLC therapeutic targets and cancer therapies are essential in improving patient survival, symptom control and quality of life. 

DNA double strand breaks (DSBs) are considered amongst the most cytotoxic lesions to cells, as their incorrect repair leads to genomic instability and cell death [19,20]. Thus, the efficient repair of DSBs is vital in order to maintain genomic stability and cellular viability. Cells use two major pathways to repair these DSBs; non-homologous-end-joining (NHEJ) and homologous recombination (HR). NHEJ is the predominant pathway and re-joins broken DNA ends without the use of extensive homology and can be used in any phase of the cell cycle. On the other hand, HR repairs DSBs with high fidelity and only occurs when a sister chromatid is available as a repair template and thus only occurs during the S or G2 cell cycle phases [21,22,23,24]. 

DNA repair enzymes are currently at the center of intense interest for the treatment of cancer, with the advent of PARP inhibitors [25]. While a cells’ DNA repair pathways prevent mutations that lead to cancer, once a tumor has developed, these DNA repair pathways can be exploited to induce cancer cell death [22]. When a tumor continually grows, DNA repair pathways become dysregulated as a result of genetic streamlining and compensatory mechanisms are activated, resulting in increased tumor adaptability to the environment [26]. Thus, inhibitors of DNA repair proteins function by disrupting compensatory mechanisms, resulting in cell death [26]. At present there are inhibitors of several DNA repair proteins under various stages of development as cancer therapeutics [26,27]. 

In recent years, a family of copper metabolism gene MURR1 domain (COMMD) proteins, have demonstrated potential as cancer therapeutics [28,29]. The COMMD family of proteins consists of ten evolutionarily conserved members (COMMD1-10) that contain a highly conserved carboxy terminal COMM domain. Numerous biological activities including copper homeostasis, the activity of the NF-κB transcription factor, cell proliferation and protein trafficking [30,31] are regulated by COMMD proteins. 

COMMD1 is the most thoroughly characterized member of the COMMD family [32,33]. A recent study demonstrated that by modulating p21 and Cip1 levels, COMMD1 regulates cell proliferation and the cell cycle [34] of HEK293 cells. The authors showed that the overexpression and knockdown of COMMD1 regulated the proliferation of HEK293 cells. Cell cycle analyses demonstrated that the overexpression of COMMD1 arrested cells in the G1 phase of the cell cycle.

Recently, COMMD4 was shown to be a novel DNA repair protein required for the timely repair of DSBs [35]. COMMD4-depleted cells were hypersensitive to DNA damaging agents and were unable to repair DSBs efficiently. In a separate publication, the authors additionally showed that *COMMD4* transcripts are upregulated in NSCLC and elevated COMMD4 was associated with poor prognosis for the ADC subtype of NSCLC [36]. Furthermore, COMMD4 protein expression was also elevated in the NSCLC cell lines. siRNA-mediated depletion of COMMD4 resulted in reduced cell proliferation and reduced cell viability of the NSCLC cells, with increased cell death after exposure to DNA damaging agents. In summary, COMMD4 depletion resulted in the NSCLC cells undergoing mitotic catastrophe and apoptosis, suggesting that COMMD4 is a good therapeutic target for the treatment of NSCLC. Similar to COMMD4, the expression of COMMD9 was also shown to be upregulated in NSCLC cells and tissues. As a result of siRNA depletion of COMMD9, inhibition of cell migration and proliferation was observed. siRNA-mediated depletion of COMMD9 also resulted in the arrest of cells at the G1/S phase of the cell cycle and autophagy induction in the NSCLC cells. COMMD9 was additionally shown to attenuate p53 signaling. Through its interaction with TFDP1, COMMD9 was found to promote TFDP1/E2F1 activation in NSCLC cells [37]. 

In this study, we have investigated the role of COMMD1 in DNA repair and its functional significance as a NSCLC diagnostic marker and therapeutic target. While COMMD1 was previously shown to interact with BRCA1, BARD1, LIG4 and CHK2 [38], its functional role in DNA repair has not been fully characterized. Here, we demonstrate a functional role for COMMD1 in the repair of DNA DSBs and further show that COMMD1 expression is upregulated in NSCLC and high *COMMD1* expression is prognostic for NSCLC patient outcome. We further show that siRNA-mediated depletion of COMMD1 markedly reduces cell proliferation and viability after exposure to DSBs induced by ionizing radiation. Taken together, we suggest COMMD1 is a novel DNA repair protein and a promising therapeutic target in NSCLC.

## 2. Materials and Methods

### 2.1. Antibodies

The following primary antibodies were used: anti-COMMD1 (Abcam, ab224727), (Invitrogen, MA5-26010), anti-β-actin (BD Biosciences, 612656), anti-gamma H2AX (Abcam, ab26350), anti-p53 Serine 15 (Cell Signaling, 9284), anti-p53 clone D0–7 (Sigma-Aldrich, p8999), anti-Chk2 Threonine 68 (Cell Signaling, 2661), anti-Chk2 (Cell Signaling, 2662), anti-ATM Serine 1981 (Cell Signaling, 13050), anti-ATM (Cell Signaling, 2873), anti-H2AX (Cell Signaling, 7631) anti-MDC1 (Abcam, 11169). The following secondary antibodies from LI-COR, Inc, were used for immunoblotting; IRDye^®^ 800CW Donkey anti-mouse (926-32212) and IRDye^®^ 680CW Donkey anti-rabbit (926-68073). The following secondary antibodies from Life Technologies were used for immunofluorescence; Alexa Fluor® 594 donkey anti-rabbit (A21207), Alexa Fluor® 594 donkey anti-mouse (A21203), Alexa Fluor® 488 donkey anti-rabbit (A21206) and Alexa Fluor® 488 donkey anti-mouse (A21202).

### 2.2. Cell Culture, Cell Treatments and Reagents

Human bronchial epithelial cells (HBEC3-KT) were cultured in keratinocyte serum-free media (Life Technologies, 17005-042, Thermo Fisher Scientific, Carlsbad, CA, USA) and 10% foetal bovine serum (FBS) (Life Technologies, 10099141, Thermo Fisher Scientific, Carlsbad, CA, USA) [39]. DDR3 cells were maintained in RPMI 1640 media (Life Technologies, 11875119) containing 10% FBS [40]. All NSCLC cells (A549, H1299, HCC827, H1975, H460, SKEMES-1, EBC-1, HTB-182, CRL-5889 and H226) were cultured in RPMI 1640 media containing 10% FBS. HBEC3-KT and NSCLC cells were cultured in a humidified incubator at 37 °C/5% CO_2_ atmosphere. The histologic features and origin of all cell lines used in this study have been previously described [36]. Hoechst 33342 was purchased from Life Technologies (H3570) and irradiations were performed at room temperature using a ^137^Cs source (Gammacell 40 Exactor [MDS Nordion]; dose rate 1.1 Gy/min).

### 2.3. Small Interfering RNA (siRNA) and Transfections

Cells were transfected with siRNA targeting COMMD1; #1 (CACCUGUUGCCAUUAUAGA[dT][dT] MISSION^®^ siRNA, Sigma-Aldrich) or COMMD1 siRNA #2 (CUGUUGCCAUUAUAGAGCU[dT][dT] MISSION^®^ siRNA, Sigma-Aldrich, St. Louis, MO, USA) to downregulate COMMD1 levels. A control siRNA (MISSION^®^ siRNA Universal Negative Control #1, Sigma-Aldrich, SIC001) was used in parallel. siRNA was transfected using RNAiMax (Life Technologies, 13778500) according to the manufacturer’s instructions and cells were analysed 48 to 72 h after transfection. Cells were transfected using FuGENE^®^ HD (Promega, E2312, Madison, WI, USA) according to the manufacturer’s instructions, with a COMMD1-FLAG siRNA-resistant plasmid (resistant to siRNA #2), which was cloned into the expression vector pcDNA3.1+/C-(K)-DYK (GenScript, Piscataway, NJ, USA). Cells were analyzed 24 h after transfection.

### 2.4. Collection of Lysates and Immunoblotting Analyses

Lysates were collected and immunoblotted as previously described [36,41]. Briefly, total cell lysates were washed with phosphate-buffered saline and lysed in ice-cold NP40 buffer (20 mM HEPES pH 8, 10 mM MgCl_2_, 150 mM KCl, 0.5 mM EDTA, 0.2% NP40, 5% glycerol, 0.5 mM DTT, 1× protease and phosphatase inhibitor cocktail (Thermo Scientific, 78444) and 1× Pierce Universal Nuclease for cell lysis (Thermo Fisher Scientific, PIE88702)). Between 10 and 15 µg of cell lysates were separated on 4–12% Bis-Tris Plus Bolt precast gels (Life Technologies, NW04120) and immunoblotted with the indicated antibodies. 

### 2.5. Quantitative Real Time PCR (qRT-PCR)

For qRT-PCR, to each well of a 384-well plate (Thermo Fisher Scientific, 43-098-49), we added 1 µl of cDNA reverse transcribed from total RNA, 50 nM of forward and reverse primer, 1× final concentration of SYBR green PCR mix (Thermo Fisher Scientific, A25742) and nuclease-free water (Thermo Fisher Scientific, 10-977-015) to make up the final reaction volume to 10 µL. qPCR was subsequently performed using a ViiA7 real-time PCR system (Life Technologies) at 95 °C for 10 min, followed by 40 cycles of 95 °C for 15 s, 62 °C for 60 s and a final primer-template dissociation step. The following primer pair was used to amplify *COMMD1* transcript; Forward Primer 5′-GCTGGAGAGTTGATGGCAAGTC-3′ and reverse primer 5′-GACCTCATCAAATTCCAAACACAG-3′), while the following primer pair was used to amplify *7SL* transcript levels; forward primer 5′-ATCGGGTGTCCGCACTAAGTT-3′ and Reverse Primer 5′-CAGCACGGGAGTTTTGACCT-3′). *COMMD1* mRNA transcript levels were normalized to *7SL* transcript levels using the comparative C_T_ method.

### 2.6. Patient Samples and Immunohistochemistry, Imaging and Analysis

Tissue microarrays (TMA) containing ADC and SCC tissue arrays were purchased (US Biomax Inc, LC808b and LC706a, Derwood, MD, USA). Immunohistochemistry was performed as previously described using the Ventana Discovery Ultra (Ventana) automated immunohistochemistry stains (Roche) [36]. For staining with anti-COMMD1 antibody, slides were incubated with anti-COMMD1 antibody which was diluted 1:100 in PBS for 1 h at ambient temperature. Multi-Spectral Images (MSI) of each core were captured on the Vectra III Spectral Scanner using 20× objective using the slide annotation from Phenochart. Analysis of MSI were performed as previously described on InForm Cell Analysis Software (PerkinElmer, Waltham, MA, USA) [36]. TMA staining intensity in parallel was evaluated by a pathologist (Cheng Liu) and assessed on a semiquantitative scale, with the score based on staining intensity; 0 = no staining, 1 = weak staining, 2 = moderate staining, 3 = strong staining. Nuclear and cytoplasmic staining were assessed separately. Scores obtained were separated by the median and correlated with the available clinicopathological parameters, including cancer type, age, sex, grade, stage and TNM Score analyzed with the Chi^2^-Test.

### 2.7. Cell Proliferation and Apoptosis Assays

For cell proliferation, 3 × 10^3^ cells were seeded into clear 96-well plates and were imaged using the Incucyte S3 (Essen BioScience, Ann Arbor MI, USA). Viable and apoptotic cells were measured using the Annexin V-FITC apoptosis kit using the methodology recommended by the manufacturer (United Bioresearch, ALX-850-020-K101, Enzo Life Sciences, Farmingdale, NY, USA). In summary, HBEC3-KT and NSCLC were resuspended in binding buffer containing annexin V-FITC. After the recommended incubation, cells were subsequently incubated with propidium iodide and flow cytometry was performed with the cytoFLEX flow cytometer (Beckman Coulter, Brea, CA, USA). The software CytExpert 2.0 was used for data acquisition and data were analyzed using the FlowJo v10 software. 

### 2.8. Clonogenic Cell Viability Assays

Clonogenic cell viability assays were carried out as previously reported [36,41]. In summary, following transfection with either control or COMMD1 siRNA #1 or #2, 500 cells for each treatment were plated in well of a 6-well plate and 24 h later, irradiated with the doses of IR indicated. For the transfection with an siRNA-resistant COMMD1 plasmid, 24 h post-transfection with siRNA, cells were transfected with the plasmid, plated on 6-well plates and irradiated the following day. Cells were allowed to recover for 8–10 days prior to counting colonies. Data are represented as means ±SD and dose-response curves were generated using Graphpad Prism 8.

### 2.9. Cell Cycle Analyses

To analyze the percentage of cells in S phase an EdU imaging assay was performed. Briefly, semi-confluent cells were grown in black 96-well plates in the presence of 10 µM EdU (Thermo Fisher Scientific, C10635) for 60 min. After fixation in 4% *w*/*v* formaldehyde, permeabilization with 0.2% Triton X-100, EdU labeling with 4 mM Sulfo-Cyanine5 azide (Lumiprobe, A3330) using click chemistry and counterstaining with 5 µg/mL of Hoechst33342, fluorescence intensities for Cyanine5 and Hoechst in single nuclei was measured using an IN Cell 6500 and IN Carta software (GE Life Sciences, GE Healthcare, Chicago, IL, USA) and the ratio of EdU-positive to total nuclei per well was determined with R software.

### 2.10. Analysis of NHEJ and HR Function Using an In Vivo Fluorescence-Based Reporter

This assay was performed as previously described [40]. In summary, U20S cells stably expressing the reporter were transfected with control, COMMD1 siRNA and BRCA1 siRNA. At 48 h post-transfection, the cells were transfected with both the *I-Sce1* and exogenous donor plasmids. Then, 48 h post-transfection of both plasmids, the cells were trypsinized and flow cytometry was performed using a cytoFLEX flow cytometer (Beckman Coulter, Brea, CA, USA) in order to assess the percentage of GFP and mCherry positive cells. BRCA1 siRNA was used as the control for HR and 10 µm of the DNA-PK inhibitor (Selleckchem, NU7441, Houston, TX, USA) was used as the control to assess NHEJ [42]. Data were analyzed using the FlowJo v10 software.

### 2.11. Immunofluorescence Microscopy

H1975 cells transfected with control or COMMD1 siRNA #1 or #2 were grown in 96 well plates (Cellvis, P96-1.5H-N, Mountain View, CA, USA). Following irradiation, H1975 cells were pre-extracted with ice-cold extraction buffer (20 mM HEPES (pH 8), 5 mM MgCl_2_, 20 mM NaCl, 1 mM ATP, 0.5% NP40) for 5 min, to remove soluble proteins and processed as previously described [41]. Images were captured using the Delta Vision PDV microscope 100×/1.42 Oil objective (Applied Precision, Inc, Issaquah, WA, USA) and the IN Cell Analyzer 6500 Imaging System (GE Healthcare Life Sciences, Chicago, IL, USA). A minimum of 50 nuclei were quantified per independent experiment and images were assembled with Adobe Photoshop CC 2019.

### 2.12. Bioinformatics and Statistical Analysis

A Kaplan–Meier plotter [43] database was used to perform *COMMD1* survival analysis as previously described [36]. For gene expression and gene set enrichment analyses, *COMMD1* expression levels in lung cancer samples were obtained from The Cancer Genome Atlas (TCGA) Illumina HiSeq 2000 RNA Sequencing platform and log2-transformed. Using the GO_DNA_REPAIR gene set, single-sample gene set enrichment analysis (ssGSEA) scores were determined as previously described [44]. Expression levels and ssGSEA scores were compared using Spearman’s rank correlation analyses.

All data are presented as the mean ±SD from ≥ three independent experiments. A two-tailed non-paired Student’s *t*-test was used for all statistical analyses. *COMMD1* mRNA and protein expression, dose-response and growth curves were compared using a paired Student’s *t*-test from ≥ three independent experiments. The level of significance was set at * *p* ≤ 0.05 and ** *p* ≤ 0.005. Statistical methods were not used to predetermine the sample size. 

## 3. Results

### 3.1. COMMD1 Is Required for the Repair of DNA DSBs

**Figure 1:**
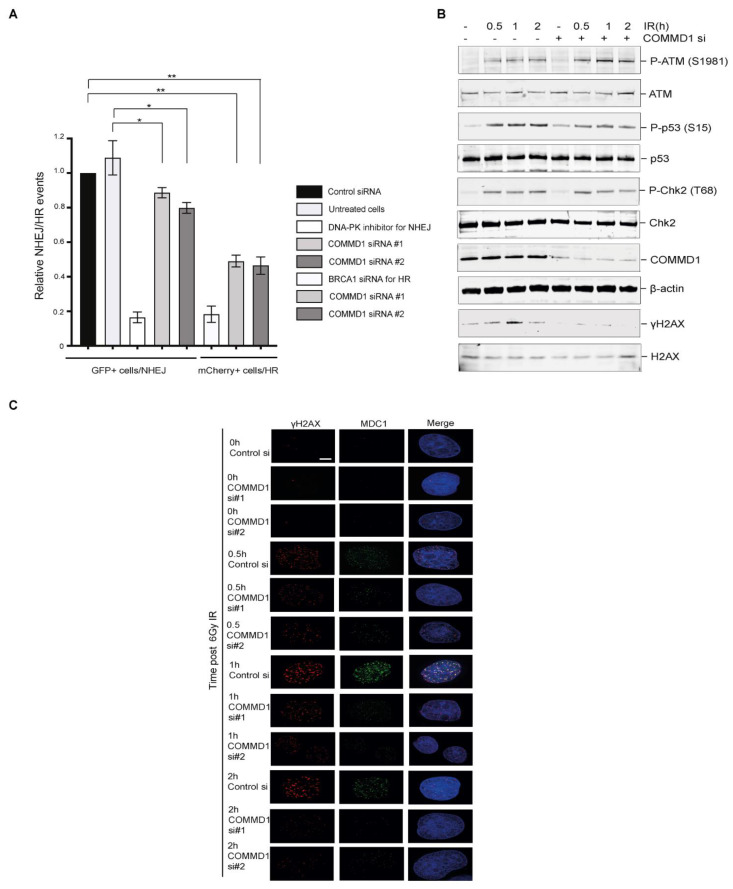
Defective DNA repair in COMMD1-depleted cells. (**A**) Plot of the relative non-homologous-end-joining (NHEJ) and homologous recombination (HR) events respectively in control, untreated, DNA-PK inhibitor, BRCA1 siRNA and COMMD1-depleted cells using a quantitative reporter assay that measures NHEJ versus HR in the same cells through the repair of two inverted ISce1 cuts. * *p* < 0.05, ** *p* < 0.005. Error bars represent mean ± S.D from three independent experiments. (**B**) DNA damage signaling in H1975 cells transfected with control and COMMD1 siRNA at 0, 0.5, 1 and 2 h post-irradiation. β-actin was used as the loading control. (**C**) Immunofluorescence experiment demonstrating γH2AX and MDC1 foci formation in control and COMMD1-depleted H1975 cells before and after irradiation. DAPI shows the nucleus. Scale bar denotes 5 µm. The uncropped Western Blot figures in Figure S4.

COMMD1 is both a nuclear and cytoplasmic protein that interacts with CHK2, BRCA1, BARD1 and LIG4 [38], suggesting a role for COMMD1 in DNA repair. In order to investigate a function for COMMD1 in the repair of DSBs, we examined the activity of NHEJ and HR, using a previously described in-cell reporter assay in stably transfected U20S cells (DDR3) [40]. This assay demonstrated that both NHEJ and HR function were defective in COMMD1-depleted cells (Figure 1A and Figure S1A) and that COMMD1 is required for a common processing step in NHEJ and HR. Upon the induction of DSBs, cell signaling events are activated which include checkpoint activation and the repair of the break [22]. H1975 NSCLC cells depleted of COMMD1 demonstrated increased activation of ATM (P-ATM 1981), however, reduced p53 S15 stabilization, reduced pChk2 T68 phosphorylation and reduced phosphorylation of H2AX (γ-H2AX), further showing defective DNA repair (Figure 1B and Figure S1B). The reduced γ-H2AX, p53 S15 and pChk2 T68 signaling may represent the down regulation of the regulatory pathways involved in their phosphorylation and dephosphorylation. An siRNA-resistant plasmid of COMMD1 (to siRNA #2) was generated to rescue the defective DNA repair phenotype in NSCLC cells depleted of COMMD1 (Figure S1C). We further examined γ-H2AX foci formation [45] and MDC1 [46] foci formation in H1975 cells (Figure 1C and Figure S1D), which corroborated our DNA damage signaling data.

### 3.2. COMMD1 Gene Transcripts Are Upregulated in NSCLC and This Upregulation Is Associated with Poor Patient Outcome

**Figure 2:**
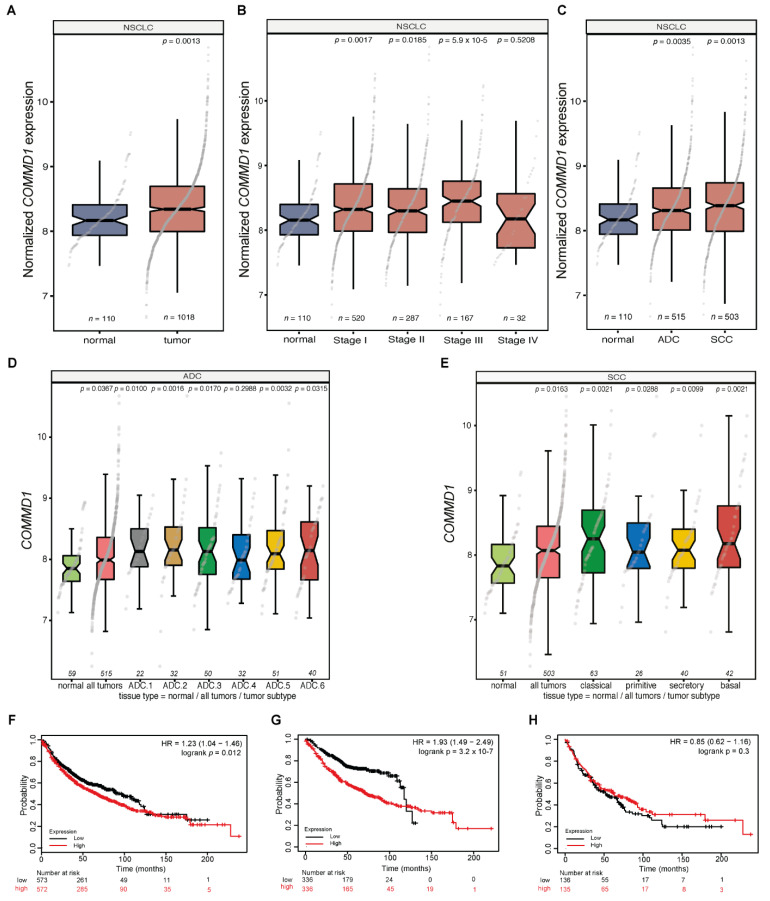
*COMMD1* gene transcripts are upregulated in non-small cell lung cancers (NSCLC) and this is associated with poor patient outcome. (**A**–**C**). Box plots of *COMMD1* transcripts comparing normal to tumor tissue (**A**), non-malignant to stages I–IV (**B**) and the comparison between the expression of *COMMD1* in adenocarcinoma (ADC) and squamous cell carcinoma (SCC) (**C**). (**D**,**E**). A boxplot of the expression of *COMMD1* in different ADC and SCC subtypes. All *p*-values in (**A–D**): Mann–Whitney U tests, compared to normal tissues. (**F**–**H**). Kaplan–Meier analysis of overall survival of 1144 NSCLC, 865 ADC and 675 SCC cases, comparing high versus low *COMMD1* expression split by median expression level. Cox proportional hazard ratio (HR), 95% confidence interval and corresponding *p*-values are shown. NSCLC; non-small cell lung carcinoma, ADC; adenocarcinoma, SCC; squamous cell carcinoma.

DNA DSBs cause physical cleavage of the DNA backbone and are among the most dangerous lesions. A single unrepaired DSB in a cell can be cytotoxic [22,47], additionally mutations or down-regulation of DNA repair proteins, associated with the repair of DSBs, are strongly linked with inherited cancer risk (BRCA1, BRCA2, ATM) [48,49,50,51]. Here, as we confirmed a role for COMMD1 in the repair of DSBs and as COMMD4 was previously shown to function in the repair of DSBs [35] and additionally a potential therapeutic target and prognostic factor in NSCLC [36], we investigated a similar role for COMMD1 in NSCLC. Initially, the expression of *COMMD1* transcripts were evaluated using bioinformatic analysis of The Cancer Genome Atlas (TCGA) datasets (Figure 2A–E). *COMMD1* transcripts were analyzed across all NSCLC stages and histologies relative to normal healthy tissue. The expression of *COMMD1* was upregulated in all stages of NSCLC (*n* = 1018) compared to non-malignant tissue (*n* = 110, *p* = 0.0013) (Figure 2A,B). Further bioinformatic analysis demonstrated that *COMMD1* expression is significantly upregulated in both the ADC and SCC subtypes of NSCLC, with the relative expression elevated in ADC (*n* = 515, *p* = 0.0035) and SCC (*n* = 503, *p* = 0.0013), compared to non-malignant tissue (Figure 2C). Furthermore, *COMMD1* expression was significantly increased in five of the six ADC subtypes (Figure 2D) and in all of the SCC subtypes (Figure 2E), compared to normal tissue. As *COMMD1* was overexpressed in NSCLC, we investigated whether the increased mRNA expression correlated with patient survival. Univariate Kaplan–Meier analysis of 1144, 865 and 675 NSCLC, ADC and SCC cases respectively, showed that patients with upregulated *COMMD1* expression showed a worse outcome than those with low *COMMD1* expression (HR = 1.23, CI: 1.04–1.46, log rank *p* = 0.012) (Figure 2F). For the further elucidation of the prognostic value of *COMMD1*, we subsequently stratified for the histological subtypes. For 865 ADC NSCLC cases, high *COMMD1* expression was found to be prognostic for patient outcome (HR = 1.93, CI: 1.49–2.49, log rank *p* = 3.2 × 10^−7^) (Figure 2G), although, patient numbers were low towards the tail of the KM plot. The analysis of 675 SCC cases showed that *COMMD1* was not prognostic (HR = 0.85, CI: 0.62–1.16, log rank *p* = 0.3) (Figure 2H) in this subtype. Taken together, these data suggest that *COMMD1* expression steadily increases during ADC disease progression, whilst *COMMD1* expression is typically continuously high during the more aggressive development of SCC.

### 3.3. COMMD1 Protein Levels Are Differentially Expressed in Tissue and Cell Lines from NSCLC Patients

The expression of COMMD1 protein was evaluated by immunohistochemistry in 74 ADC and 78 SCC, both in the form of patient TMA’s. Of the 74 ADC NSCLC cases, none exhibited COMMD1 staining in the nucleus and 67 of 74 (90%) cases exhibited staining solely in the cytoplasm; with 3% of cases exhibiting weak staining (staining intensity 1), 45% with moderate (staining intensity 2) staining and 45% with strong (staining intensity 3) staining. Of the 78 SCC, 8 of 78 cases (10%) exhibited COMMD1 staining solely in the nucleus; all 10% with weak (staining intensity 1) staining and 8 of 78 SCC cases (10%) exhibited staining solely in the cytoplasm; 76% with weak (staining intensity 1) staining and 10% with strong (staining intensity 3) staining (Figure S2 and Table 1). Intensity scores were stratified by median score and matched with clinicopathological parameters. High nuclear staining of COMMD1 correlated with SCC NSCLC (*p* = 0.0136) relative to ADC NSCLC, whereas lower cytoplasmic COMMD1 staining was associated with SCC NSCLC (*p* = 2.18 × 10^−22^) relative to ADC NSCLC. Lower COMMD1 expression in the cytoplasm was also associated with males (*p* = 1.36 × 10^−6^) in contrast to females (Figure S2 and Table 1). Nonetheless, we did not find a significant correlation between either nuclear or cytoplasmic staining of COMMD1 and age, surgical stage tumor grade or the TNM score. 

**Figure 3:**
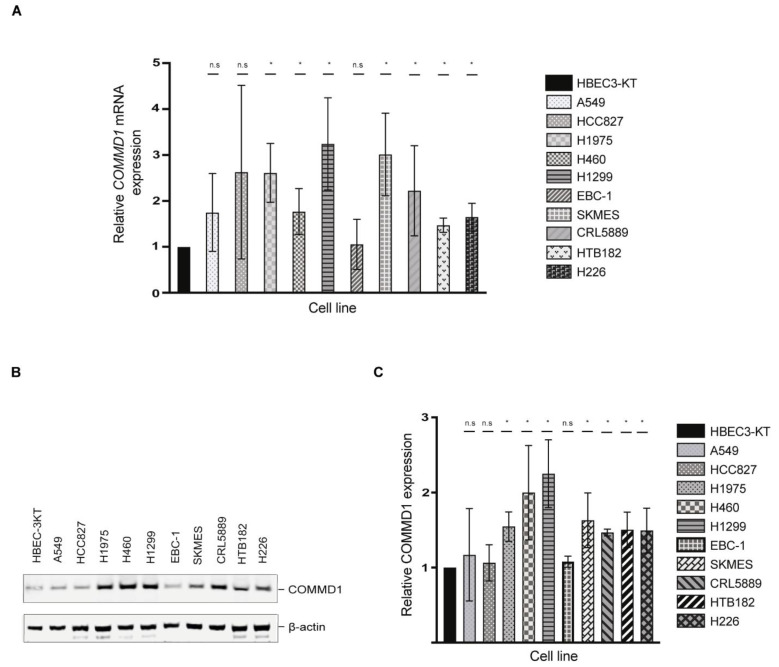
COMMD1 mRNA and protein expression in NSCLC cells. (**A**) qRT-PCR analysis of *COMMD1* transcript in the immortalized epithelial cell line (HBEC3-KT) and ten NSCLC cell lines, relative to the *7SL* housekeeping gene and relative to the HBEC3-KT cells. (**B**) An immunoblot showing the expression of COMMD1 protein in lysates from the HBEC3K-T and the ten NSCLC cells. β-actin indicates the loading. (**C**) The quantification of the levels of COMMD1 protein relative to actin and then relative to HBEC3K-T cells (Figure 3B) is shown. * *p* < 0.05. n.s; not significant. Error bars represent mean ± S.D from three independent experiments. The uncropped Western Blot figures in Figure S5.

*COMMD1* mRNA transcript levels and protein expression were next assessed in the immortalized non-tumorigenic HBEC3-KT cell line and in 10 NSCLC cell lines. qRT-PCR analyses showed that *COMMD1* mRNA levels are significantly elevated in 7 out of 10 of the NSCLC cells in comparison to the HBEC3-KT cells (Figure 3A). In the ADC (H1975 and H1299), SCC (SKMES, CRL5889, HTB 182 and H226) and LCC (H460) cell lines, we observed significantly higher levels of *COMMD1* relative to HBEC3-KT cells. We next performed immunoblotting analyses with HBEC3-KT and the 10 NSCLC cell lines to elucidate if *COMMD1* transcript correlates with COMMD1 protein expression. In line with our qRT-PCR data, immunoblotting experiments demonstrated that all the cell lines with elevated *COMMD1* transcript; namely H1975, H460, H1299, SKMES, CRL5889, HTB182 and H226 also showed higher expression of COMMD1, relative to HBEC3-KT cells (Figure 3B,C).

### 3.4. COMMD1 Is Required for NSCLC Proliferation and Cell Cycle Progression

**Figure 4:**
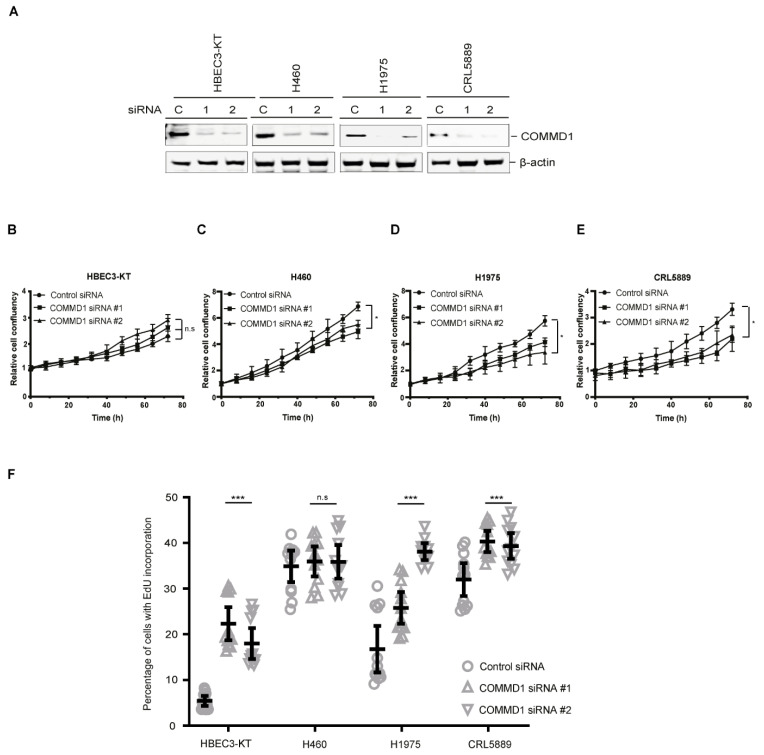
COMMD1 is required for proliferation of NSCLC cells. (**A**) Immunoblot showing siRNA-mediated depletion of COMMD1 with control siRNA or COMMD1 siRNA #1 (1) or #2 (2) across the HBEC3-KT and three NSCLC cell lines. β-actin shows the loading. (**B**–**E**) Proliferation analysis of HBEC3-KT, H460, H1975 and CRL5889 cells depleted of COMMD1 with control or siRNA #1 or #2 and analysed using the Incucyte S3 live imaging system. Asterix (*) denotes *p* < 0.05. n.s; not significant. Error bars represent mean ± SD from three independent experiments. (**F**) Cell cycle analysis of the percentage of cells with EdU incorporation in HBEC3-KT and NSCLC cells. Cells were depleted with control siRNA and COMMD1 siRNA #1 and #2. ANOVA and Tukey’s multiple comparison test was used to evaluate the portion of S phase cells in control vs COMMD1-depleted cells. * *p* < 0.05, *** *p* < 0.0005 and n.s; not significant. Error bars represent mean ± 95% CI, *n* = 12 from three independent experiments. The uncropped Western Blot figures in Figure S6.

As COMMD1 is overexpressed in NSCLC lung cancer cell lines, we employed two different siRNA sequences (#1 and #2) that target the *COMMD1* transcript, as well as a negative control siRNA to deplete COMMD1 from HBEC3-KT, H460, H1975 and CRL5889 cell lines, as they represent the bronchial epithelial, LCC, ADC and SCC subtypes respectively (Figure 4A). Depletion of COMMD1 levels by siRNA was evaluated by immunoblotting of cell lysates. Relative to the control siRNA, COMMD1 siRNA #1 and #2 depleted the expression of COMMD1 by approximately 80–90% (Figure 4A). We next assessed whether COMMD1 depletion affected NSCLC cell proliferation using the Incucyte S3. While we observed no significant change in growth of HBEC3-KT cells after siRNA depletion of COMMD1 (Figure 4B), in the NSCLC cell lines, there was a significant retardation of cell growth after COMMD1 depletion with siRNA #1 and #2 (Figure 4C–E), suggesting that COMMD1 is required for the proliferation of NSCLC cells. To further explore the mechanism of how COMMD1 depletion results in retardation of NSCLC cell growth, we analyzed cell cycle progression in COMMD1-depleted control, H460, H1975 and CRL5889 NSCLC cell lines. Cell cycle analyses demonstrated that COMMD1-depletion resulted in a significant increase in the S-phase of HBEC3-KT, H1975 and CRL5889 cells (Figure 4F). These data demonstrate that the observed increase in the portion of cells in S phase is a consequence of COMMD1 depletion and not specific to NSCLC.

### 3.5. Depleting COMMD1 Sensitises NSCLC Cells to Irradiation

**Figure 5:**
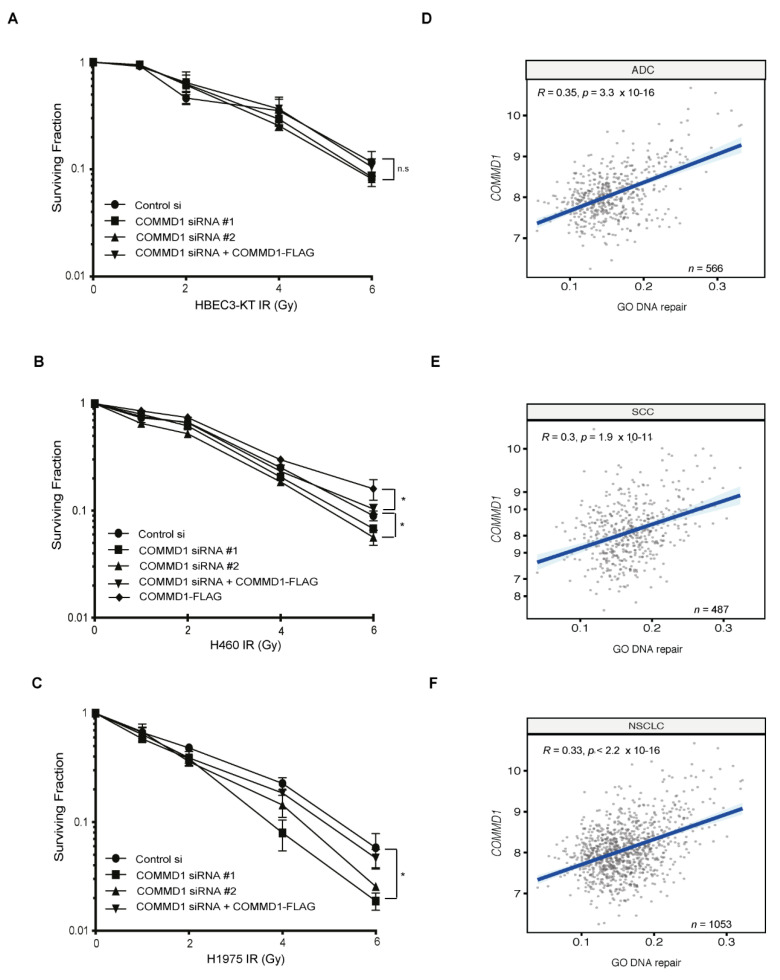
COMMD1 is required for the survival of NSCLC cells after exposure to irradiation and measures of COMMD1 DNA repair in NSCLC. (**A**–**C**). Clonogenic cell viability assays in HBEC3-KT, H460 and H1975 NSCLC cells transfected with control or COMMD1 siRNA (#1 or #2) and treated with varying doses of irradiation (IR). Correction of the IR defect in cells depleted of COMMD1 (with siRNA #2) using a COMMD1 siRNA-resistant plasmid and overexpression of COMMD1-FLAG is also shown. Asterix (*) denotes *p* < 0.05. n.s; not significant. Error bars represent mean ± SD from three independent experiments. (**D**–**F**). Correlation between *COMMD1* expression levels and the gene ontology (GO) DNA repair single-sample gene set enrichment analysis (ssGSEA) scores in lung adenocarcinoma (ADC), lung squamous cell carcinoma (SCC) and non-small cell lung cancer (NSCLC) samples from The Cancer Genome Atlas (TCGA). R and *p*-values: Spearman’s rank correlations.

As knockdown of COMMD1 was previously shown to sensitize breast cancer cells to the DNA damaging agents, cisplatin and doxorubicin [38], COMMD4 depletion sensitizes NSCLC cells to irradiation [36] and COMMD1 functions in the repair of DSBs (Figure 1), we explored whether COMMD1-depleted NSCLC cells were hypersensitive to irradiation. For the treatment of NSCLC, radiotherapy is used in early-stage patients who are medically inoperable, as well as in stage IIIA and IIIB patients as a frontline therapy [11], [52,53]. We initially assessed the therapeutic potential of depleting COMMD1 on radiation sensitivity. Here, clonogenic cell viability assays were utilized [54] to assess if COMMD1 knockdown in combination with ionizing radiation may result in reduced viability of NSCLC cells, relative to NSCLC cells treated with control siRNA. While the HBEC3-KT cells did not display hypersensitivity to ionizing radiation (Figure 5A), COMMD1-depleted H460 and H1975 cells displayed hypersensitivity (Figure 5B,C). The overexpression of an siRNA-resistant plasmid of COMMD1 rescued the hypersensitivity to irradiation in H460 and H1975 cells (Figure S1C and Figure 5A–C), while overexpression of COMMD1-FLAG alone, showed radiation resistance (Figure 5B). Taken together, this shows that COMMD1 depletion enhances the sensitivity of NSCLC cells to radiation and demonstrates a functional role for COMMD1 in protecting cells from DSBs induced by irradiation, further corroborating a role for COMMD1 in the repair of DSBs.

As COMMD1 siRNA depletion of NSCLC cells resulted in reduced cell proliferation and reduced cell viability after exposure to ionizing radiation, we thus assessed whether COMMD1 depletion additionally led to apoptosis. Flow cytometry analysis of propidium iodide and Annexin V staining demonstrated that depleting cells of COMMD1 did not result in the significant induction of apoptosis in NSCLC cells compared to the HBEC3-KT cells (Figure S3).

### 3.6. Bioinformatic Analyses of Genomic Instability

As we demonstrated that COMMD1 depletion resulted in increased hypersensitivity to irradiation and COMMD1-depleted cells are defective in the repair of DSBs by both NHEJ and HR, we used bioinformatic analyses to determine whether *COMMD1* levels correlate with measures of DNA repair (Figure 5D–F). *COMMD1* expression showed a positive correlation with the gene ontology (GO) DNA repair gene set enrichment signature in lung cancer samples. We observed a positive correlation between *COMMD1* expression and ADC, SCC and NSCLC. This data further corroborates that COMMD1 functions in DNA repair. 

## 4. Discussion

COMMD proteins containing a C-terminal COMM domain are novel potential anti-cancer therapeutic targets and prognostic markers [30,31,35]. The expression of COMMD4 [36] and COMMD9 were previously assessed in NSCLC [37]. 

As COMMD1 has previously been shown to interact with CHK2, BRCA1, BARD1 and LIG4 [38], we explored whether COMMD1 functioned in the repair of DSBs. We have previously shown a role for COMMD4 in the repair of DSBs [36]. Here, we used a reporter assay to measure the repair of DSBs by NHEJ and HR in control and COMMD1-depleted cells. This assay suggests that both NHEJ and HR DSB repair are functionally impaired in the absence of COMMD1. While COMMD1-depletion only resulted in a 10–20% reduction in NHEJ, COMMD1 has previously been shown to interact with LIG4 [38], a protein involved in the process of NHEJ [55], which further corroborates our data and suggests a role for COMMD1 in NHEJ. In this study we demonstrated a significant reduction in HR in the absence of COMMD1 as well as defective DNA damage signaling and impaired DNA repair foci with the depletion of COMMD1. This data, together with an interaction with CHK2 and BRCA1 [38], highlights a role for COMMD1 in HR. COMMD1 has previously been shown to interact with other COMMD proteins, especially COMMD3, 4 and 6 [31]. As COMMD4 has been shown to directly function in DNA repair by regulating H2B monoubiquitination [35], it is likely that COMMD1 functions in a complex with COMMD4 to regulate this process. 

Once we established a role for COMMD1 in DNA repair we next studied the role of COMMD1 in NSCLC. The meta-analysis of *COMMD1* transcripts demonstrated that NSCLC patients had significantly higher levels of *COMMD1* than control normal tissue, irrespective of the NSCLC subtype or the tumor grade. Patients with ADC and high *COMMD1* expression also exhibited a poorer prognosis relative to patients who had lower *COMMD1* expression. qRT-PCR analysis corroborated our bioinformatic data, where the majority of NSCLC cell lines demonstrated significantly higher expression of *COMMD1* transcript relative to the HBEC3-KT bronchial epithelial cell line. The *COMMD1* expression observed in NSCLC cell lines did not correlate with EGFR mutation status, where H460 and A549 express wild-type EGFR, while H1975 express a L858R and T790M mutation [56,57]. To determine whether *COMMD1* transcript levels correlated with COMMD1 protein levels, we performed immunoblotting of the ten NSCLC and control bronchial epithelial cell line. Our immunoblotting experiments corroborated the qRT-PCR as well as clinical in vitro data, where COMMD1 protein levels were statistically upregulated in the majority of NSCLC assessed, relative to the HBEC3-KT cell line. *COMMD1* transcript and protein expression showed statistical significance in most, however, not in every NSCLC cell line, that may be attributed to HBEC3-KT cells adapting to its culture environment.

TMA analysis of NSCLC patient samples corroborated our mRNA and immunoblotting analyses, where COMMD1 levels were high irrespective of age, tumor grade, surgical stage or the TNM score. Nevertheless, we observed statistically higher nuclear COMMD1 protein expression in SCC patients relative to ADC patients, while lower COMMD1 cytoplasmic staining was associated with SCC compared to ADC and lower COMMD1 levels in the cytoplasm was associated with males relative to females. However, in our COMMD1 TMA analysis, patient survival data was not included, a larger data set with patient outcome being required to further confirm the prognostic potential of COMMD1 protein levels in NSCLC.

As *COMMD1* transcript and protein overexpression is seen in NSCLC, we next examined whether this overexpression was essential for the survival of NSCLC cells. siRNA-mediated depletion of COMMD1 was established with two independent siRNA sequences targeting COMMD1. Transfection of control and the H460, H1975 and CRL5889 NSCLC cells demonstrated that COMMD1 targeting siRNA considerably reduced the expression of COMMD1 in these cells and moreover, significantly reduced the proliferation of the NSCLC cells compared to the control HBEC3-KT cell line. These observations suggest that COMMD1 plays a role in the proliferation of NSCLC cell lines. 

With advances in targeted radiotherapy and medical imaging, radiotherapy remains a frontline option for treatment of NSCLC [58]. In the immunotherapy era, there are currently numerous studies combining conventional radiotherapy with immune checkpoint inhibitors for NSCLC [59]. In this study, we assessed the viability of NSCLC cells depleted of COMMD1, in combination with irradiation, using clonogenic cell viability assays. We observed that COMMD1-depleted NSCLC cells were hypersensitive to irradiation relative to the NSCLC cells transfected with control siRNA. In contrast, the HBEC3-KT cell line depleted of COMMD1 did not demonstrate hypersensitivity to irradiation. Taken together, these results suggest that COMMD1 is required for the survival of these NSCLC cells after induction of radiation-induced DNA damage. As irradiation in combination with COMMD1 depletion demonstrated a synergistic effect in NSCLC cells, we hypothesize that COMMD1 depletion in combination with DNA damage inducing agents may present as a treatment option for NSCLC. There has been some disparity in the field regarding the sensitivity of COMMD1-depleted cancer cells to DNA damaging agents. COMMD1 depletion was previously shown to increase cell viability, while overexpression of COMMD1 reduced the viability [34] of HEK293 cells. Another study showed that elevated nuclear expression of COMMD1 in ovarian cancer cells resulted in cisplatin sensitivity and led to the accumulation of cells in S phase. Furthermore, the authors also observed increased cisplatin-induced apoptosis in these cells [60]. In contrast, [38] showed that depletion of COMMD1 resulted in hypersensitivity of breast cancer cells to the DNA damaging agents’ cisplatin and doxorubicin. Here we show that COMMD1 depletion in combination with irradiation, reduces the viability of NSCLC cells. HEK293 cells are human embryonic kidney cells [61] and differences observed by [34] and by [60] may be attributed to the different origin of the cell types. 

Although COMMD1 depletion reduced the proliferation and viability of NSCLC cells, COMMD1 depletion did not result in significant apoptosis of these cells. We additionally assessed cell cycle progression in COMMD1-depleted cells. COMMD1 was previously shown to regulate the cell cycle in HEK293 cells [34]. The authors demonstrated that COMMD1 depletion by shRNA resulted in a decrease in cells in the G1 phase of the cell cycle. Their data additionally demonstrated a trend towards an increase in S and G2 cells in the COMMD1-depleted HEK293 cells, although this did not reach statistical significance. Interestingly, [37] demonstrated that siRNA-mediated depletion of COMMD9 in NSCLC cells resulted in reduced proliferation and migration of these cells, arrested cells at the G1/S phase of the cell cycle and additionally induced autophagy of the NSCLC cells. In our study, we observed increased S phased cells after COMMD1 depletion, indicating a DNA repair defect associated with aberrant mitotic progression when COMMD1 is depleted from cells. This is in line with a previous study that showed that COMMD1 controls cell cycle progression by regulating p21 Cip1 levels [34]. Depletion of COMMD1 in H460 cells did not affect the number of cells in S phase, however, reduced the proliferation of these cells. This is in contrast to the H1975 and CRL5889 NSCLC cells lines, where COMMD1 depletion resulted in increased S phased cells and reduced proliferation. Based on other studies, this difference observed may be attributed to the varying genetic background between each of the cell lines, which may affect their observed cell cycle progression through S phase [62]. These observations warrant further investigation to determine the impact of COMMD1 expression during the cell cycle.

PARP inhibitors are clinical cancer therapeutics that function by targeting HR deficiency [63]. CRISPR screens were performed to elucidate genes and pathways that lead to resistance to the PARP inhibitor, olaparib [64]. The authors demonstrated that *COMMD1* was amongst 73 genes where mutations cause increased sensitivity to olaparib. The authors identified several DNA repair genes regulating HR, such as *BRCA1, *BARD1*,* *BRCA2* and *PALB2*, as well as the *ATM* and *ATR* kinases. *COMMD1* has been previously shown to interact with BRCA1 and BARD1 [38]. These observations combined with our observation that COMMD1 is required for the efficient repair of DSBs via HR, support a functional role for COMMD1 in HR and raises the intriguing possibility that patients harboring inactivating mutations of COMMD1 may be sensitive to PARP inhibitors.

## 5. Conclusions

In summary, our study suggests that COMMD1 functions in the repair of DSBs and that COMMD1-deficient cells have failure of both NHEJ and HR DSB repair pathways. Compared with normal tissue, *COMMD1* mRNA and protein levels are overexpressed in NSCLC patients and *COMMD1* functions as a prognostic marker for the ADC subtype of NSCLC. In NSCLC cell lines, COMMD1 was found to promote cancer cell proliferation and COMMD1 depletion by siRNA significantly decreased cell proliferation and reduced NSCLC cell viability. Taken together, we show that COMMD1 is a prognostic marker and a novel therapeutic target in NSCLC, functioning to repair DSBs through NHEJ and HR. This study highlights a novel avenue in the treatment of NSCLC, by targeting a new player in the DNA repair cascade. 

## Figures and Tables

**Table 1:** Association of COMMD1 tissue microarrays (TMA) scores with clinicopathological features.

Characteristic	(Nuclear) Total, *n*	(Nuclear) Low (<=Median), *n*	(Nuclear) High (>Median), *n*	(Nuclear) Chi *p*-value	(Cytoplasmic) Total, *n*	(Cytoplasmic) Low (<=Median), *n*	(Cytoplasmic) High (>Median), *n*	(Cytoplasmic) Chi *p*-Value
**Histological Type**								
ADC	74	74	0	0.0136	74	7	67	2.18 × 10^−22^
SCC	78	70	8		78	70	8	
**Age**								
Age <= 65	109	104	5	0.8485	109	57	52	0.6440
Age > 65	43	40	3		43	20	23	
**Sex**								
Male	113	105	8	0.2052	113	71	42	1.36 × 10^−6^
Female	38	38	0		38	6	32	
**Tumour Grade**								
Grade 1	11	11	0	0.6618	11	8	3	0.2362
Grade 2	58	54	4		58	26	32	
Grade 3	72	68	4		72	36	36	
**Surgical Stage**								
Stage IA–IB	54	52	2	0.5719	54	30	24	0.5755
Stage IIA–IIB	61	58	3		61	28	33	
Stage IIIA–IIIB	34	31	3		35	17	18	
**TNM Score**								
TNM-T (1–2)	119	112	7	0.8347	119	64	55	0.2055
TNM-T (3–4)	33	32	1		33	13	20	
TNM-N 0	67	65	2	0.3663	67	36	31	0.0560
TNM-N 1	65	61	4		65	27	38	
TNM-N 2	18	16	2		18	13	5	

ADC; adenocarcinoma, SCC; squamous cell carcinoma, TNM; tumor, node, metastasis.

## Data Availability

All data presented in this study are included within the paper and its Supplementary files.

## Supplementary Materials

The following are available online at https://www.mdpi.com/2072-6694/13/4/830/s1, Figure S1. Quantification of COMMD1 depletion and overexpression, Figure S2. COMMD1 expression in NSCLC patient TMA’s, Figure S3. COMMD1 depletion does not result in apoptosis of NSCLC cells, Figure S4. Uncropped Western Blot Images for Figure 1B, Figure S5. Uncropped Western Blot Images for Figure 3B, Figure S6. Uncropped Western Blot Images for Figure 4A, Figure S7. Uncropped Western Blot Images for Figure S1A, Figure S8. Uncropped Western Blot Images for Figure S1C.
