# Supplementary material for: COMMD1, from the Repair of DNA Double Strand Breaks, to a Novel Anti-Cancer Therapeutic Target
Source: Cancers (Basel). 2021 Feb 16;13(4):830. doi: 10.3390/cancers13040830 (PMC7920454; doi:10.3390/cancers13040830)
Supplement: Supplementary file 1 [file cancers-13-00830-s001.pdf]

Supplementary Materials: COMMD1, from the Repair of DNA Double Strand Breaks, to a Novel Anti-Cancer Therapeutic Target

Amila Suraweera, Pascal H.G. Duijf, Christian Jekimovs, Karsten Schrobback, Cheng Liu, Mark N. Adams, Kenneth J. O'Byrne and Derek J. Richard

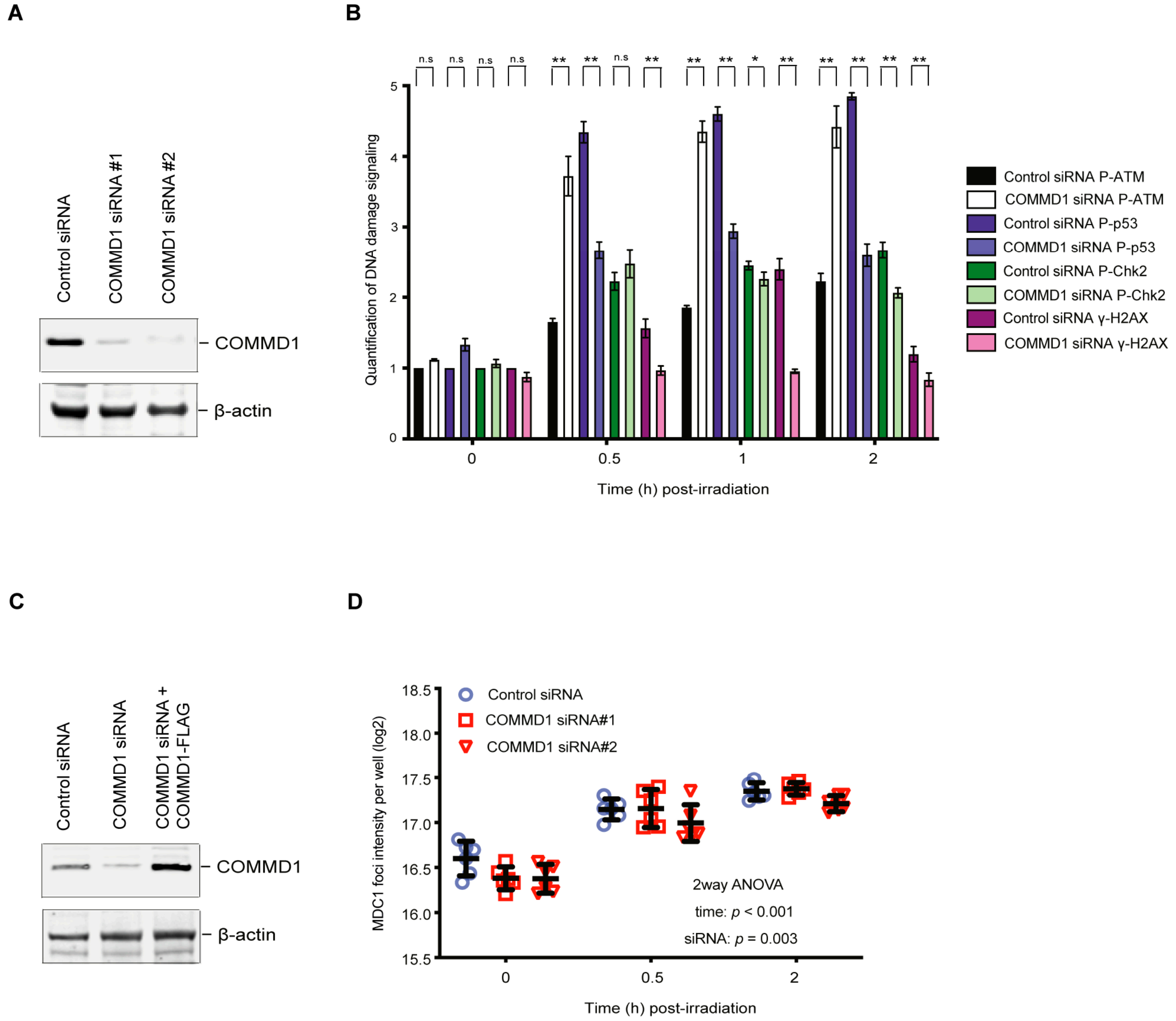

Figure S1. Quantification of COMMD1 depletion and overexpression. (**A**) An immunoblot showing the downregulation of COMMD1 for Figure 1A. (**B**) Quantification of the DNA damage signalling in control and COMMD1 depleted cells after treatment with ionizing radiation for Figure 1B. Levels of P-ATM/p53/Chk2/γH2AX were calculated relative to total ATM/p53/Chk2/H2AX respectively. (**C**) An immunoblot showing the depletion of COMMD1 using control siRNA or COMMD1 siRNA #2 and overexpression of cells with an siRNA-resistant COMMD1 (siRNA #2) plasmid. β-actin shows the loading. (**D**) Quantification of MDC1 foci intensity for control and COMMD1 siRNA #1 and #2 treated cells from Figure 1C, using 2way ANOVA. n.s; not significant, *; $p < 0.05$, **; $p < 0.005$. Error bars represent mean ± S.D from three independent experiments. The uncropped Western Blot figures in Figure S7 and Figure S8.

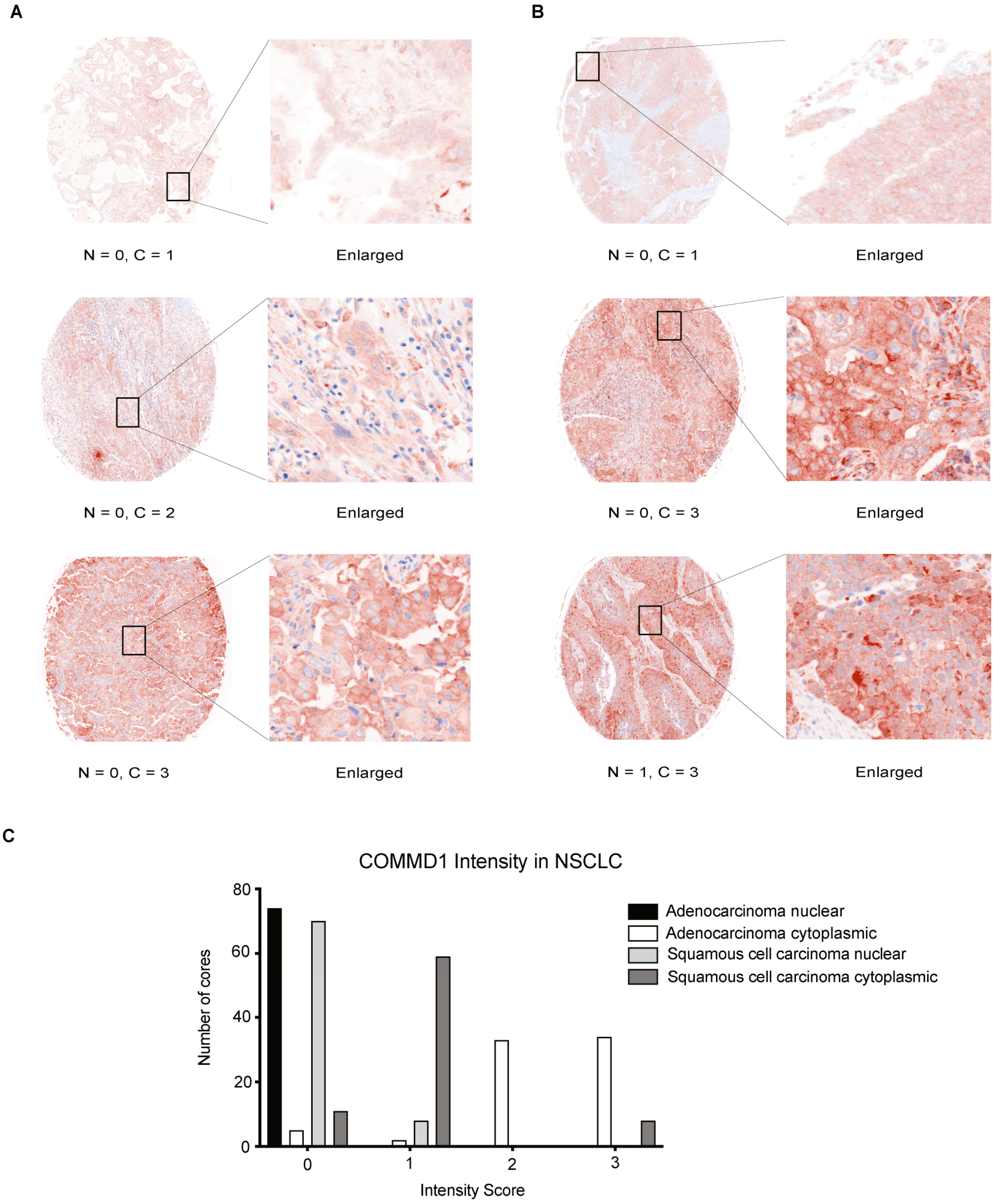

Figure S2. COMMD1 expression in NSCLC patient TMA's. (**A**) and (**B**) Representative images of negative (0), weak (1), moderate (2) and strong (3) scores for COMMD1 nuclear and cytoplasmic staining of adenocarcinoma (**A**) and squamous cell carcinoma (**B**) TMAs. N; nuclear, C; cytoplasmic. (**C**) Graph showing COMMD1 nuclear and cytoplasmic staining intensity of adenocarcinomas and squamous carcinomas across all TMA cores.

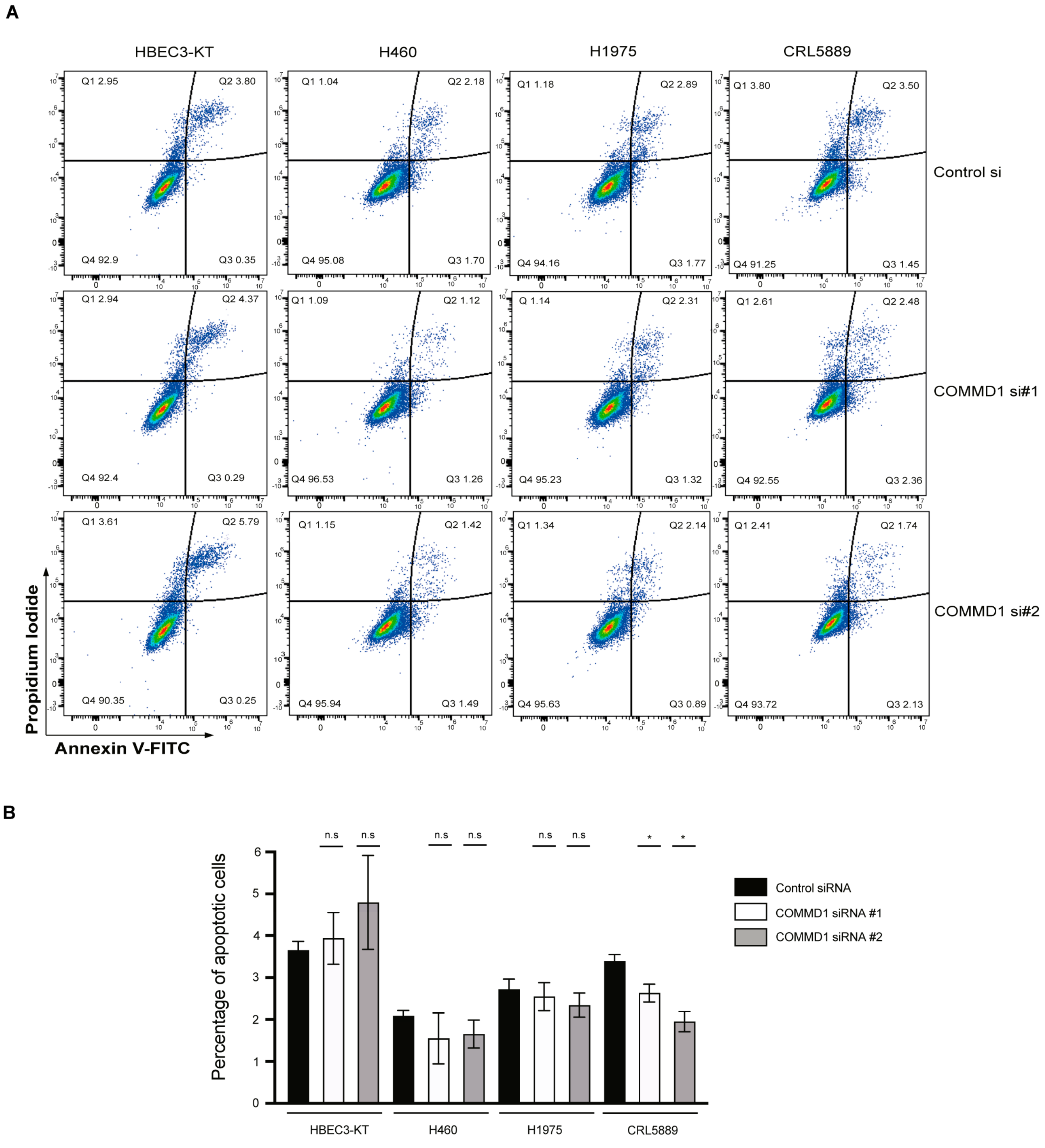

Figure S3. COMMD1 depletion does not result in apoptosis of NSCLC cells. (**A**) Flow cytometry analyses of apoptosis with control siRNA and siRNA #1 and siRNA #2 treated HBEC3-KT, H460, H1975 and CRL5889 cell lines. Live cells were stained with Annexin V-488 and propidium iodide and assessed using a CytoFLEX flow cytometer. The percentage of cells within each quadrant is listed for each panel. Live cells; lower left quadrant (Q4), apoptotic cells; right quadrants (Q2 and Q3). (**B**) Quantification of (**A**). The percentage of apoptotic cells is shown and was calculated from the quadrants Q2+Q3. n.s; not significant, *; $p < 0.05$. Error bars represent mean ± S.D from three independent experiments.

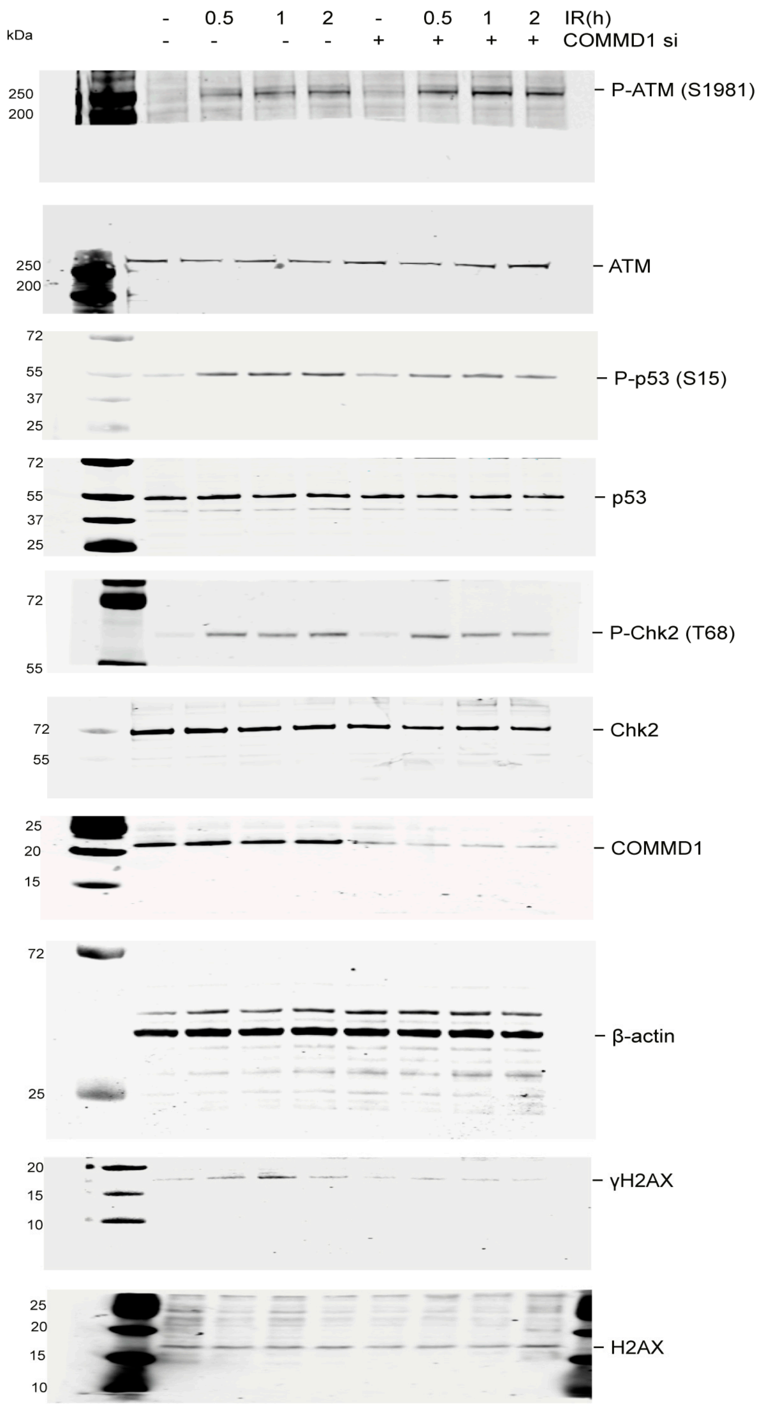

Figure S4. Uncropped Western Blot Images for Figure 1B.

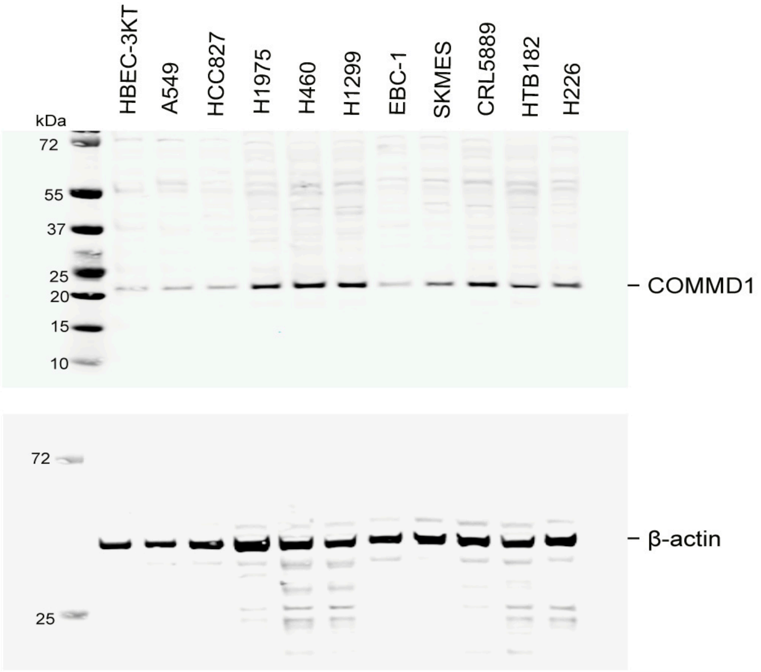

Figure S5. Uncropped Western Blot Images for Figure 3B.

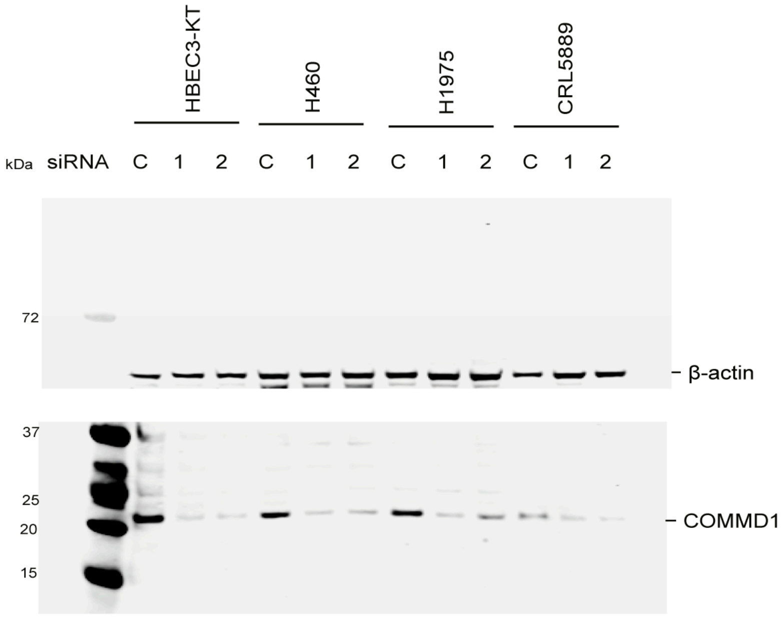

Figure S6. Uncropped Western Blot Images for Figure 4A.

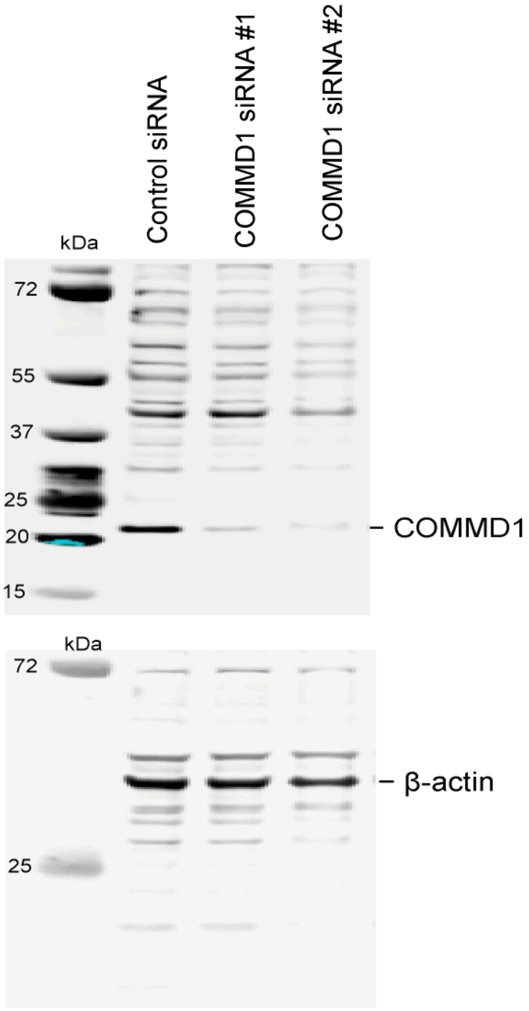

Figure S7. Uncropped Western Blot Images for Figure S1A.

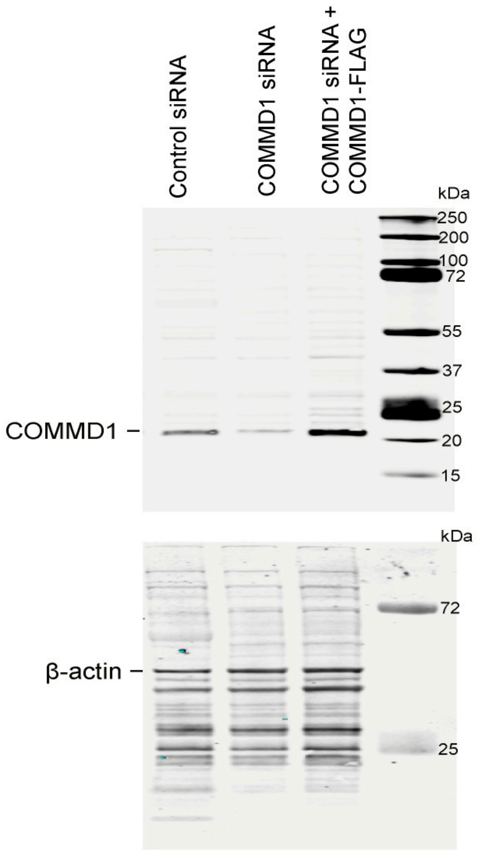

Figure S8. Uncropped Western Blot Images for Figure S1C.

Copyright: © 2021 by the authors. Licensee MDPI, Basel, Switzerland. This article is an open access article distributed under the terms and conditions of the Creative Commons Attribution (CC BY) license (http://creativecommons.org/licenses/by/4.0/).
